# Employment and Economic Outcomes of Participants With Mild Traumatic Brain Injury in the TRACK-TBI Study

**DOI:** 10.1001/jamanetworkopen.2022.19444

**Published:** 2022-06-29

**Authors:** Étienne Gaudette, Seth A. Seabury, Nancy Temkin, Jason Barber, Anthony M. DiGiorgio, Amy J. Markowitz, Geoffrey T. Manley

**Affiliations:** 1 Institute of Health Policy, Management and Evaluation, Dalla Lana School of Public Health, University of Toronto, Toronto, Ontario, Canada; 2 Leonard D. Schaeffer Center for Health Policy & Economics, University of Southern California, Los Angeles; 3 School of Pharmacy, University of Southern California, Los Angeles; 4 Department of Neurological Surgery, University of Washington, Seattle; 5 Department of Biostatistics, University of Washington, Seattle; 6 Department of Neurological Surgery, University of Washington, Seattle; 7 Department of Neurological Surgery, University of California, San Francisco; 8 Zuckerberg San Francisco General Hospital, San Francisco, California

## Abstract

**Importance:**

Mild traumatic brain injury (mTBI) may impair the ability to work. Strategies to facilitate return to work are understudied.

**Objective:**

To assess employment and economic outcomes for employed, working-age adults with mTBI in the 12 months after injury and the association between return to work and employer assistance.

**Design, Setting, and Participants:**

Using data from the Transforming Research and Clinical Knowledge in Traumatic Brain Injury (TRACK-TBI) study, a cohort study of patients with mTBI presenting to emergency departments of 11 level I US trauma centers was performed. Patients with mTBI enrolled in the TRACK-TBI cohort study from February 26, 2014, to May 4, 2016, were followed up at 2 weeks and 3, 6, and 12 months after injury. Work status and income decline of participants were documented in the first year after injury. Associations between work status, injury characteristics, and offer of employer assistance and associations between follow-up care and employer assistance were investigated. Results were adjusted for unobserved outcomes using inverse probability weighting. Data were extracted July 12, 2020; analyses were completed March 24, 2021. Analyses included 435 participants aged 18 to 64 years who were working before the injury, had a Glasgow Coma Scale score of 13 to 15, and completed all postinjury follow-up surveys.

**Main Outcomes and Measures:**

Primary outcomes were work status (working or not working) at each study follow-up milestone. Employer assistance included sick leave, reduced hours, modified schedule, transfer to different tasks, assistive technology, and coaching offered during the first 3 months after injury.

**Results:**

Of 435 participants (147 [34%] female; 320 [74%] White; mean [SD] age 37.3 [12.9] years), 258 (59%) reported not working at 2 weeks after injury and 74 (17%) reported not working at 12 months after injury. More than one-fifth (92 [21%]) experienced a decline in annual income. Work status at 12 months was significantly associated with postconcussion symptoms experienced at 3 months after injury (73% of patients with 3 or more symptoms reported working at 12 months after injury vs 89% of patients with 2 or fewer symptoms; *P* < .001) but not with other injury characteristics. Participants offered employer assistance in the first 3 months after injury were more likely to report working after injury than those not offered such assistance (at 6 months: 88% vs 78%; *P* = .02; at 12 months: 86% vs 72%; *P* = .005).

**Conclusions and Relevance:**

In this cohort study, mTBI was associated with substantial employment and economic consequences for some patients. Clinicians should systematically follow up with patients with mTBI and coordinate with employers to promote successful return to work.

## Key Points

**Question:**

What are the employment and economic outcomes of patients with mild traumatic brain injury?

**Findings:**

In this cohort study of 435 previously employed study participants with mild traumatic brain injury, more than half (59%) reported not working at 2 weeks after injury; 17% reported not working at 12 months after injury. Those who were offered employer assistance in the first 3 months after injury were significantly more likely to report working at 12 months.

**Meaning:**

This study found that mild traumatic brain injury is associated with substantial employment and economic consequences for some patients; clinician engagement to support early access to employer assistance could help moderate the economic burden for patients.

## Teaser

This cohort study examines employment and economic outcomes and the association between employer assistance and return to work 12 months after mild traumatic brain injury (TBI) among participants in the TRACK-TBI Study.

## Introduction

Mild traumatic brain injury (mTBI) may have ongoing consequences for functional outcomes and disability in the postacute period. Recent reports from the multi-institutional research initiative Transforming Research and Clinical Knowledge in TBI (TRACK-TBI) Study have revealed long-term sequelae of mTBI: 12 months after mTBI, 53% of participants displayed some functional limitation.^1^ Even in patients with mTBI who had no evidence of abnormalities on computed tomography scans, 35% showed some functional disability at 6 months.^2^ Other studies have also confirmed a range of consequences after mTBI, including high rates of functional disability, risk for psychiatric problems,^3^ difficulty with executive functioning,^4^ and dementia.^5^

Along with worsening functional outcomes, it has also been found that many patients with mTBI (8%-26%) are unable to work at 3 to 6 months after injury.^6,7,8,9^ Worse employment outcomes are associated with postconcussion symptoms,^7,8,10,11^ lower levels of education,^6,7,8,10^ and younger age.^9,10^

Although negative associations of mTBI and employment outcomes are clear, treatments and strategies to facilitate successful return to work (RTW) have not been widely studied. Previous research has largely focused on specific symptom-based therapies to facilitate RTW (eg, use of vestibular therapies to address dizziness).^12,13^ However, RTW is a multifactorial process involving complex interactions among clinical and social/environmental factors, in the face of which symptom-based interventions alone could be inadequate. A strategy that warrants further scrutiny is assistance provided by employers in the workplace. In a recent study^14^ of factors perceived to facilitate RTW, 76% of study participants who successfully returned to work after mild to moderate TBI identified employer support as helpful. However, the repercussions of such assistance for the proportion of patients with mTBI who return to work and the types of assistance with the greatest impact have yet to be assessed.

Using data from the prospective, multicenter, longitudinal observational TRACK-TBI cohort study, we documented the work status of previously employed working-age adults with mTBI in the first year after injury. We also measured the proportion of participants reporting a decline in income at 12 months after injury. We investigated associations between working at 6 months and 12 months after injury with baseline injury characteristics, early postconcussion symptoms, and employer assistance. Then, we examined whether participants who saw a health care professional in the 3 months after an injury were more likely to be offered employer assistance.

## Methods

### Study Data

#### Participants and Data Collection

The TRACK-TBI Study enrolled patients with TBI who were first seen in the emergency departments of 11 level I US trauma centers (eTable 1 in Supplement 1). Each enrolling institution obtained institutional review board approval. Adult participants with TBI enrolled between February 26, 2014, and May 4, 2016, were eligible for this analysis. Participants or their legal representatives provided written informed consent within 24 hours of injury. The Strengthening the Reporting of Observational Studies in Epidemiology (STROBE) reporting guideline was followed. (eFigure 1 in Supplement 1 shows participant recruitment and retention flow.) Inclusion criteria for the TRACK-TBI Study included acute head trauma sufficient for an emergency department physician to order a clinical head computed tomography scan within 24 hours of injury. Mild traumatic brain injury was defined as a patient presenting with a Glasgow Coma Scale (GCS) score of 13 to 15, loss of consciousness for less than 30 minutes, and/or posttraumatic amnesia duration less than 24 hours, in agreement with the American Congress of Rehabilitation Medicine’s criteria.^15^ Exclusion criteria were pregnancy, incarceration, nonsurvivable physical trauma, debilitating mental health disorders (eg, severe unmedicated schizophrenia, poorly managed bipolar disorder) or neurologic disease (eg, Alzheimer disease), and preexisting medical conditions that could interfere with outcome assessments. This cohort study restricted the analysis to patients with mTBI aged 18 to 64 years who were employed at the time of the injury and who answered the employment status question at all milestones. At each milestone (2 weeks and 3, 6, and 12 months after injury), participants were followed up with standard outcome assessments and a 200-question interview to assess follow-up medical care as well as the economic and health consequences of TBI. All data used in the analysis were self-reported by participants or surrogates with the exception of the GCS score, which was the result of a clinical assessment. Data were extracted July 12, 2020; analyses were completed March 24, 2021.

#### Primary Outcomes

The primary goal of the analysis was to examine participants’ work status after injury and how it varied according to injury characteristics and assistance offered by employers. Primary outcomes of the study were work status at each study milestone after injury. The following baseline and early injury characteristics were included in the analysis: GCS score on arrival at the emergency department, admission to a hospital ward or intensive care unit, and moderate to severe postconcussion symptoms at 2 weeks and 3 months after injury. Postconcussion symptoms were assessed with the Rivermead Post Concussion Symptoms Questionnaire.^16^ This questionnaire asks respondents about the severity of problems experienced compared with problems experienced before the injury, including headache, dizziness, and nausea as well as cognitive, mood, and sleep disturbances and other physical symptoms associated with postconcussion syndrome. Symptoms were rated on a scale of 0 to 4, with 0 indicating the symptom was not experienced at all and 4 indicating the symptom was a severe problem. For purposes of the TRACK-TBI study, a 7-day observation period was used. Using the methods of Sterr et al,^17^ we considered participants to have postconcussion syndrome if they reported 3 or more postconcussion symptoms with a severity rating of 3 or higher. Participants were asked whether any of the following 6 types of employer assistance had been offered: sick leave, part-time or reduced hours, modified schedule, transfer to a different occupation with different tasks, equipment or assistive technology to help perform the job, or coaching or mentorship.

#### Secondary Outcomes

Secondary outcomes evaluated were changes in annual income measured by self-reported pretax annual income from wages or salary, tips, commissions, bonuses, business, or practice before injury and 12 months after injury. We analyzed whether receipt of follow-up care, such as having seen a physician or any health care professional (eg, general practitioner, brain injury or concussion specialist, neurologist, physiatrist, chiropractor, psychiatrist, psychologist, alternative medicine practitioner, or other type of professional specified by the participant) for the mTBI during the first 3 months after injury, was associated with being offered employer assistance.

### Statistical Analysis

#### Descriptive Analysis

We conducted a descriptive analysis of employment status dynamics after injury. We graphically presented the distribution of self-reported employment status before injury and at 2 weeks and 3, 6, and 12 months after injury. We then compared the proportion of participants reporting a lower annual income level at 12 months after injury by working status. Specifically, we compared participants who reported not working at 12 months with the overall study population, with participants who reported working at 12 months, and with participants who reported working in all follow-up surveys (“worked continuously through 12 months”).

We then assessed the association between study variables and the likelihood of working. We examined the association between early injury characteristics and employment status at 12 months. Next, we estimated the association between being offered employer assistance during the first 3 months after injury and the likelihood of reporting working 6 months and 12 months after injury. We compared rates of employer assistance offered to participants who saw and those who did not see a health care professional by 3 months after injury.

#### Weighting

Excluding participants based on lack of follow-up can introduce bias into an analysis because those unobserved outcomes are often associated with the factors that allowed them to be unobserved. Inverse probability weighting can help account for this by deriving a set of weights to effectively make the cohort being analyzed more closely resemble the original cohort and then applying these weights when analyzing outcomes. In this analysis, we used a boosted regression algorithm to assess the propensity for participants to have complete employment status information at all TRACK-TBI assessment points (2 weeks, 3 months, 6 months, and 12 months after injury). Weights were then calculated by inverting the modeled propensities for those with a complete employment status record and then rescaling such that the mean weight in the sample remained equal to 1. Sample sizes reported are weighted values rounded to the nearest integer, and percentages are calculated from unrounded weighted samples. The corresponding unweighted versions are provided in eFigures 2, 3, and 4 and eTables 4, 5, and 6 in Supplement 1.

#### Testing

Participant characteristics (eTable 2 in Supplement 1) were assessed for statistical imbalance before and after weighting. To minimize assumptions regarding underlying distributions, Mann-Whitney tests were used for continuous and ordinal variables, and Fisher exact tests were used for nominal variables. Differences in injury characteristics and postconcussion symptoms (eTable 3 in Supplement 1) and employer assistance were assessed for statistical significance using Fisher exact tests after controlling for a 5% false discovery rate per the Benjamini-Hochberg (BH) correction.^18^ All statistical testing used a 2-sided significance threshold of *P* < .05.

The boosted regression for assessing the inverse probability weighting was carried out using the Toolkit for Weighting and Analysis of Nonequivalent Groups application.^19^ All other statistical analyses were conducted using IBM SPSS Statistics for Windows, version 26 (IBM Corp).

## Results

Of 749 working-age participants with mTBI who were employed before injury, 435 answered the employment status question at all follow-up milestones and were included in this analysis. The mean (SD) age of included participants was 37.3 (12.9) years; 147 participants (34%) were female, 320 (74%) were White, and 363 (84%) were non-Hispanic. A total of 352 participants (81%) worked full-time before their injury, and 230 (53%) reported an annual income of less than $50 000. eTable 2 in Supplement 1 describes the characteristics of the analyzed sample and those excluded because employment status was unknown for at least 1 follow-up milestone. Excluded participants were more likely to be White, Hispanic, working less than full-time before injury, less educated, injured in a car accident, and admitted to the intensive care unit.

**Figure 1.**
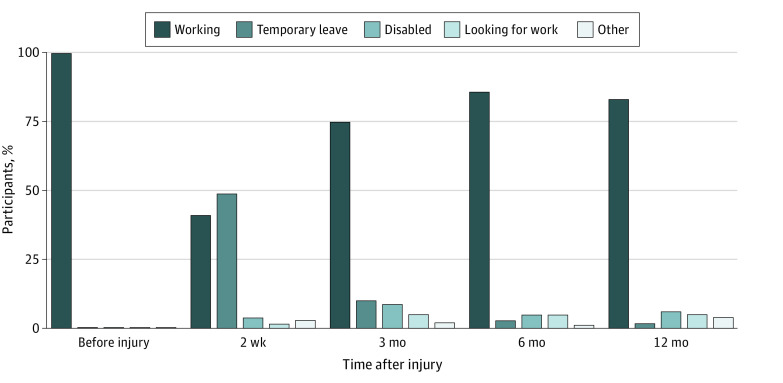
Employment Status of Participants Before Injury and Through 12 Months After Injury Weighted analysis. Temporary leave status is defined as not working due to health issues (including maternity leave) but having a job to return to; disabled status is defined as not working due to health issues and not having a job to return to; and other status includes keeping house, being a student, being retired, having an unknown status, and mentioning an unlisted status.

At 2 weeks after injury, 258 participants (59%) reported not working. Of those, 214 (83%) reported being on temporary leave, and 19 (7%) reported being disabled (Figure 1). The largest increase in RTW occurred between 2 weeks and 3 months after injury, after which RTW rates increased modestly. At 12 months after injury, 74 participants (17%) reported not working. One hundred fifty participants (34%) reported working at all milestones.

**Figure 2.**
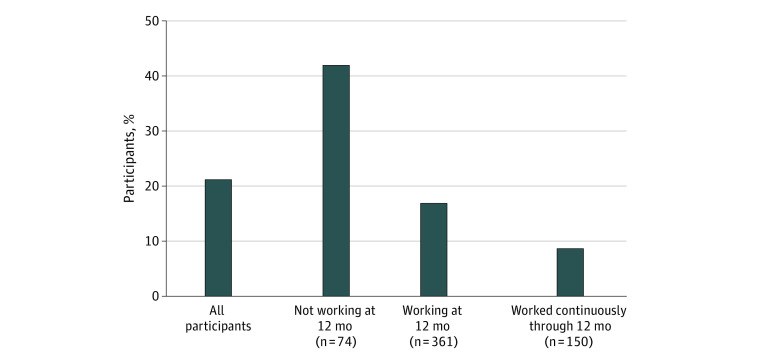
Proportion of Participants Reporting Annual Income Decline by Employment Status 12 Months Weighted analysis. Participants who “worked continuously through 12 months” are the subset of participants reporting working at 12 months who also reported working at 2 weeks, 3 months, and 6 months after injury.

Time out of work after injury was associated with a decline in income for patients with mTBI. Ninety-two participants (21%) reported an income decline in the year following their mTBI (Figure 2). Participants who reported not working at the 12-month milestone were significantly more likely to report a decline in income than those who reported working (42% vs 17%; *P* < .001). Among the 361 participants who reported working at 12 months, the 150 participants who reported working at all follow-up interviews were less likely to report a decline in income than the 211 who reported not working in at least 1 previous milestone (9% vs 23%; *P* < .001).

Although baseline injury characteristics considered in the analyses (GCS score and hospital admission after presentation to the emergency department) were not significantly associated with working at 12 months after the injury, postconcussion symptoms were associated with employment status (Table 1). At 2 weeks after injury, there was a nonsignificant association between reporting more than 3 moderate to severe symptoms and working at 12 months after injury. However, by 3 months after injury, there was a significant association between reporting more than 3 moderate to severe symptoms and working at 12 months after injury: participants who reported more than 3 symptoms at 3 months after injury had a 16–percentage point lower likelihood of working than those who reported fewer or no symptoms (73% vs 89%; *P* < .001 after BH correction). Associations between individual symptoms experienced at 3 months after injury and the likelihood of not working 12 months after injury are presented in eTable 3 in Supplement 1.

**Table 1.** Employment Status at 12 Months After Injury by Baseline Injury Characteristics and Postconcussion Symptoms^a^

Characteristic	No.	Working at 12 mo			
		No. (%)	Difference (95% CI), percentage points	*P* value	BH *P* value
All participants	435	361 (83)	NA	NA	NA
GCS score					
13-14	97	77 (80)	5 (−3 to 14)	.44	.55
15	338	284 (84)			
Admitted					
No	159	136 (86)	−4 (−11 to 4)	.35	.55
Yes	276	225 (82)			
Moderate or severe symptoms at 2 wk					
0-2	232	200 (86)	−7 (−15 to 0)	.05	.14
≥3	198	156 (79)			
Moderate or severe symptoms at 3 mo					
0-2	276	245 (89)	−16 (−24 to −8)	<.001	<.001
≥3	159	116 (73)			

Abbreviations: BH, Benjamini-Hochberg; GCS, Glasgow Coma Scale; NA, not applicable.

^a^ Sample sizes reported are weighted values rounded to the nearest integer, and percentages were calculated from unrounded weighted samples. Statistical significance was determined by Fisher exact test; BH indicates to *P* value after controlling for 5% false-discovery rate per Benjamini and Hochberg.^18^

As shown in Table 2, 287 of 391 participants (73%) reported being offered at least 1 form of employer assistance in the first 3 months after injury. Patients who were offered any form of employer assistance were 10 percentage points more likely to report working at 6 months (88% vs 78%; *P* = .02 after BH correction) and 14 percentage points more likely to report working at 12 months (86% vs 72%; *P* = .005 after BH correction) after injury than those who were not offered such assistance.

**Table 2.** Employment Status at 6 and 12 Months After Injury by Employer Assistance Offered^a^

Assistance offered by 3 mo	No.	Working at 6 mo				Working at 12 mo			
		No. (%)	Difference (95% CI), percentage points	*P* value	BH *P* value	No. (%)	Difference (95% CI), percentage points	*P* value	BH *P* value
All participants	435	374 (85)	NA	NA	NA	361 (83)	NA	NA	NA
Any assistance									
No	104	81 (78)	10 (2 to 20)	.01	.02	75 (72)	14 (5 to 24)	.002	.005
Yes	287	253 (88)				247 (86)			
Sick leave									
No	171	144 (84)	2 (−5 to 9)	.67	.71	132 (78)	8 (1 to 16)	.03	.05
Yes	218	188 (86)				188 (86)			
Modified schedule									
No	232	185 (80)	15 (9 to 21)	<.001	<.001	180 (77)	14 (5 to 24)	.001	.002
Yes	157	149 (95)				143 (91)			
Part-time or reduced hours									
No	247	199 (81)	14 (9 to 19)	<.001	.001	190 (77)	15 (8 to 21)	<.001	.001
Yes	143	135 (95)				132 (92)			
Transfer									
No	354	300 (85)	11 (−6 to 28)	.15	.22	287 (81)	19 (3 to 35)	.007	.02
Yes	26	25 (96)				26 (100)			
Equipment or assistive technology									
No	263	225 (86)	−5 (−16 to 25)	.44	.60	216 (82)	−4 (−24 to 17)	.71	.71
Yes	13	11 (81)				10 (78)			
Coaching or mentoring									
No	266	228 (86)	−3 (−23 to 18)	.65	.71	220 (83)	1 (−15 to 17)	.69	.71
Yes	9	8 (84)				8 (84)			
>1 Type									
No	239	193 (81)	12 (5 to 18)	.001	.003	184 (77)	14 (7 to 21)	<.001	.001
Yes	152	141 (93)				139 (91)			

Abbreviations: BH, Benjamini-Hochberg; NA, not applicable.

^a^ Sample sizes reported are weighted values rounded to the nearest integer, and percentages were calculated from unrounded weighted samples. Table rows are restricted to the 274 to 392 participants who answered each employer assistance question. Not all participants answered all questions; 2 questions (Equipment or assistive technology and Coaching or mentoring) were added to the questionnaire during the Transforming Research and Clinical Knowledge in Traumatic Brain Injury (TRACK-TBI) study. Statistical significance was determined by Fisher exact test; BH indicates the *P* value after controlling for 5% false-discovery rate per Benjamini and Hochberg.^18^

Regarding type of assistance offered, a modified schedule and part-time or reduced hours were significantly associated with working at 6 months after injury (95% of participants offered a modified schedule reported working 6 months after injury vs 80% of those not offered this form of assistance [*P* < .001 after BH correction]; 95% of participants offered reduced hours reported working vs 81% of those not offered this assistance [*P* = .001 after BH correction]). An offer of a modified schedule, part-time or reduced hours, or a transfer to a position with different tasks in the first 3 months after injury were all associated with a significantly higher probability of working at 12 months after injury. At 12 months after injury, 91% of participants offered a modified schedule reported working vs 77% of those not offered this form of assistance (*P* = .002 after BH correction); 92% of participants offered part-time or reduced hours reported working vs 77% of those not offered this form of assistance (*P* < .001 after BH correction); and 100% of participants offered a transfer reported working vs 81% of those not offered this form of assistance (*P* = .02 after BH correction). Also, participants who reported being offered more than 1 type of assistance were 12 percentage points more likely to report working at 6 months than those offered no or only 1 type of assistance (93% vs 81%; *P* = .003 after BH correction) and 14 percentage points more likely to report working at 12 months after injury than those who reported being offered no or only 1 type of assistance (91% vs 77%; *P* = .001 after BH correction).

**Figure 3.**
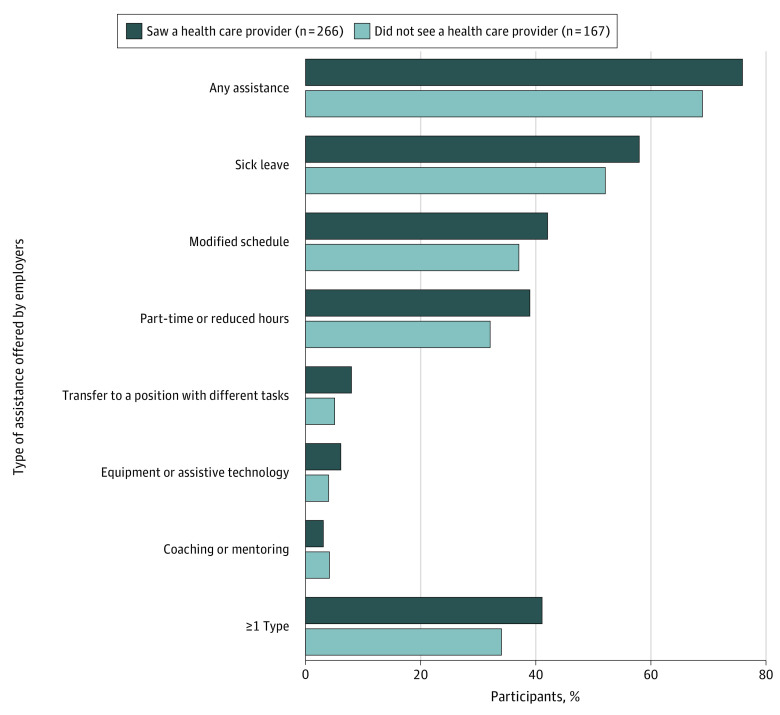
Proportion of Participants Offered Employer Assistance by Having Seen a Health Care Professional by 3 Months After Injury Weighted analysis. Health care professionals included general practitioners, brain injury or concussion clinic specialists, neurologists, physiatrists, chiropractors, psychiatrists, psychologists, alternative medicine practitioners, or any other professional specified by the participants.

Figure 3 shows the proportion of participants who reported being offered employer assistance according to whether they saw a health care professional in the 3 months following their injury. Point estimates suggest that a higher percentage of participants who saw a health care professional were offered employer assistance, although none of these differences reached statistical significance. Of those who received follow-up care, 76% were offered at least 1 form of employment assistance, 7 percentage points more than participants who did not see a health care professional (76% vs 69%; *P* = .16). Similar differences in the point estimates were found for sick leave (58% vs 52%); modified schedule (42% vs 37%); part-time or reduced hours (39% vs 32%); transfer to a position with different tasks, equipment, or assistive technology (6% vs 4%); and more than 1 type of assistance offered (41% vs 34%).

## Discussion

In the multisite, longitudinal TRACK-TBI study, fewer than half of previously employed, working-age study participants with mTBI had returned to work by 2 weeks after their injury, and 17% reported not working at 12 months after injury. Approximately 1 in 5 participants (21%) reported a decline in annual income. As in previous studies,^8,10,11^ we found that having postconcussion symptoms was inversely associated with working. We also found that patients with mTBI who were offered accommodations by their employers were more likely to be working at 6 and 12 months after injury.

Our results validate and extend previous findings that mTBI is associated with employment loss and economic burdens for patients.^6,8,9,10,11,20^ New to this study is the finding that this association may evolve over time. Symptoms reported at 2 weeks were not significantly associated with later employment; however, symptoms experienced at 3 months displayed significant association. This indicates that, for many patients, there could be a need for ongoing monitoring and symptom management even months after the injury. Given that previous work demonstrated a significant lack of follow-up with a health care professional, even for patients with a finding of an abnormality consistent with TBI on a head computed tomography scan,^21^ current treatment practices are inadequate for many patients.

To our knowledge, this study is the first to report a positive association between employer assistance and likelihood of working at 6 months and 12 months after an mTBI. In particular, being offered a modified schedule, part-time or reduced hours, or a transfer to a position with different tasks in the first 3 months after injury was associated with a higher probability of working at 12 months after injury. Although not previously reported specifically for patients with mTBI, this finding is consistent with studies demonstrating that workplace accommodations are associated with more rapid RTW for workers injured on the job.^22,23^ We also noted that participants who received follow-up care from a health care professional appeared more likely to be offered employer assistance, but the difference was not large enough to be statistically significant. The finding that the difference was not larger could reflect a critical lack of awareness of the RTW difficulties experienced by individuals with mTBI and the potential protective effect of employer support. Future research featuring a larger sample of patients should further explore this finding and investigate whether the type of professional seen and the timing of follow-up are associated with workplace assistance and RTW.

More generally, our results highlight the persisting need to develop integrated standards for follow-up care after mTBI and concussion, as recommended in a recent report by the National Academies of Sciences, Engineering, and Medicine.^24^ Improved follow-up could lead to better symptom management and help patients regain functional status, which should translate into improved ability to work and lessen the economic burden, particularly for those with postconcussion syndrome. Moreover, follow-up care professionals in an integrated system of care (eg, internists, family practice clinicians, and social workers) should engage, at a minimum, in interventions such as education that normalize the difficulties of RTW in the first weeks to months for some patients and may coordinate with employers, affirming that the patient was cared for and confirm a period of incapacity or job-related restrictions. This assistance may help to promote appropriate workplace accommodations for those experiencing persistent symptoms.

### Limitations

This study has limitations. The sample size, although larger than those of most longitudinal studies of patients with mTBI, constrained the power of the statistical analyses. The generalizability of the findings is limited to patients who reported to level I trauma centers with mTBI and required a computed tomography scan. The small number of study sites and the exclusion of participants who did not complete all follow-up surveys may also limit generalizability despite the inverse probability weighting adjustment to account for unobserved outcomes. All outcomes and most variables included in the analyses were self-reported by each patient or a surrogate and may thus be subject to biases associated with self-reporting. Participants may have had an impaired ability to recall employer assistance offered and follow-up care received in previous months. In some cases, participants may not have returned to work by 2 weeks after physician recommendation to confirm functional status at follow-up. Although the present study focused on participants with mTBI, it did not isolate the effects of head trauma exclusive of other forms of trauma. The finding that employment was more closely associated with postconcussion symptoms at 3 months compared with baseline injury characteristics suggests that mTBI symptoms were a primary factor when employment status was reported as not working. Analyses were observational and cannot establish a causal link between study variables and outcomes. Studies using experimental or quasi-experimental designs should be conducted to confirm the causality of the associations found in our study. We cannot rule out the influence of uncollected or unadjusted confounders on the apparent associations.

## Conclusions

Sustaining an mTBI is associated with substantial employment and income difficulties for some patients. This cohort study found a positive association between employer assistance and the likelihood of working at 6 months and 12 months after mTBI. Health care professionals should be more systematic about patient follow-up and recommend employer assistance, such as a modified schedule and reduced hours, to promote successful RTW for patients with concussions, especially those experiencing postconcussion symptoms.

## Notes

Supplement 1. **eTable 1.** Study Sites **eFigure 1.** Recruitment and Retention Flow Chart **eTable 2.** Participants’ Characteristics **eTable 3.** Symptoms at 3 Months by Work Status at 12 Months **eFigure 2.** Employment Status of Participants Pre-Injury and in the 12 Months After Injury (Unweighted) **eFigure 3.** Proportion of Participants Reporting Annual Income Decline by Work Status 12 Months After Injury (Unweighted) **eTable 4.** Work Status 12 Months After Postinjury by Baseline Injury Characteristics and Postconcussion Symptoms (Unweighted) **eTable 5.** Work Status 6 and 12 Months After Injury by Employer Assistance Offered (Unweighted) **eFigure 4.** Employer Assistance Offered to Participants by 3 Months After Injury by Having Seen a Healthcare Provider (Unweighted) **eTable 6.** Symptoms at 3 Months by Work Status at 12 Months (Unweighted)

Supplement 2.
Nonauthor Collaborators
