# Supplementary material for: Employment and Economic Outcomes of Participants With Mild Traumatic Brain Injury in the TRACK-TBI Study
Source: JAMA Netw Open. 2022 Jun 29;5(6):e2219444. doi: 10.1001/jamanetworkopen.2022.19444 (PMC9244609; doi:10.1001/jamanetworkopen.2022.19444)
Supplement: Supplement 1. — eTable 1. Study Sites eFigure 1. Recruitment and Retention Flow Chart eTable 2. Participants’ Characteristics eTable 3. Symptoms at 3 Months by Work Status at 12 Months eFigure 2. Employment Status of Participants Pre-Injury and in the 12 Months After Injury (Unweighted) eFigure 3. Proportion of Participants Reporting Annual Income Decline by Work Status 12 Months After Injury (Unweighted) eTable 4. Work Status 12 Months After Postinjury by Baseline Injury Characteristics and Postconcussion Symptoms (Unweighted) eTable 5. Work Status 6 and 12 Months After Injury by Employer Assistance Offered (Unweighted) eFigure 4. Employer Assistance Offered to Participants by 3 Months After Injury by Having Seen a Healthcare Provider (Unweighted) eTable 6. Symptoms at 3 Months by Work Status at 12 Months (Unweighted) [file jamanetwopen-e2219444-s001.pdf]

Supplementary Online Content

Gaudette É, Seabury SA, Temkin N, et al; TRACK-TBI Investigators. Employment and economic outcomes of participants with mild traumatic brain injury in the TRACK-TBI study. *JAMA Netw Open*. 2022;5(6):e2219444. doi:10.1001/jamanetworkopen.2022.19444

eTable 1. Study Sites

eFigure 1. Recruitment and Retention Flow Chart

eTable 2. Participants' Characteristics

eTable 3. Symptoms at 3 Months by Work Status at 12 Months

eFigure 2. Employment Status of Participants Pre-Injury and in the 12 Months After Injury (Unweighted)

eFigure 3. Proportion of Participants Reporting Annual Income Decline by Work Status 12 Months After Injury (Unweighted)

eTable 4. Work Status 12 Months After Postinjury by Baseline Injury Characteristics and Postconcussion Symptoms (Unweighted)

eTable 5. Work Status 6 and 12 Months After Injury by Employer Assistance Offered (Unweighted)

eFigure 4. Employer Assistance Offered to Participants by 3 Months After Injury by Having Seen a Healthcare Provider (Unweighted)

eTable 6. Symptoms at 3 Months by Work Status at 12 Months (Unweighted)

This supplementary material has been provided by the authors to give readers additional information about their work.

© 2022 Gaudette É et al. *JAMA Network Open*.

eTable 1. Study sites

Facility	Location
Ben Taub General Hospital	Houston, Texas
Harborview Medical Center	Seattle, Washington
Massachusetts General Hospital	Boston, Massachusetts
Parkland Memorial Hospital	Dallas, Texas
R Adams Cowley Shock Trauma Center	Baltimore, Maryland
Ryder Trauma Center	Miami, Florida
Seton Medical Center	Austin, Texas
University of Cincinnati Medical Center	Cincinnati, Ohio
University of Pittsburgh Medical Center	Pittsburgh, Pennsylvania
Virginia Commonwealth University Medical Center	Richmond, Virginia
Zuckerberg San Francisco General Hospital	San Francisco, California

© 2022 Gaudette É et al. *JAMA Network Open*.

eFigure 1. Recruitment and Retention Flow Chart

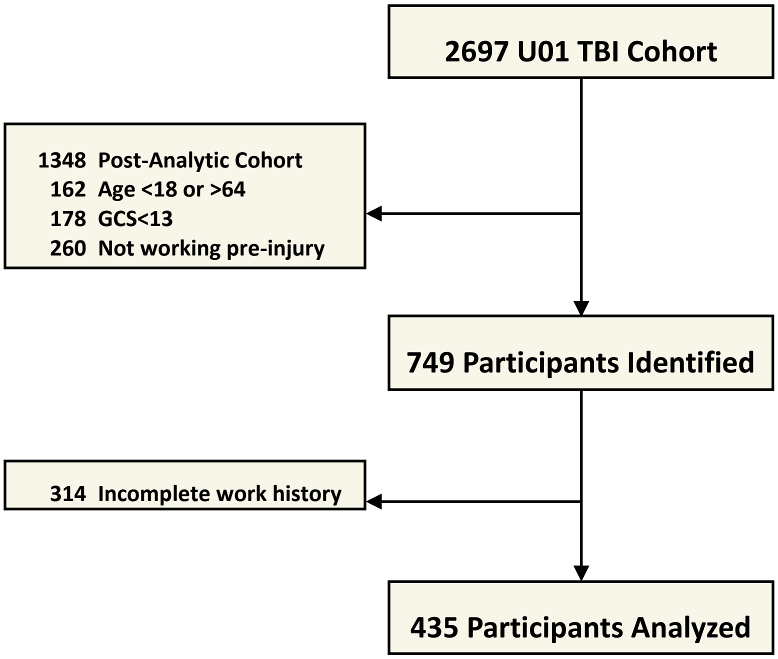

Note: U01, Full sample of the NINDS-funded TRACK-TBI study; TBI, traumatic brain injury; GCS, Glasgow Coma Scale; Analytic Cohort: cohort enrolled February 26, 2014 - May 4, 2016 and followed at 2 weeks and 3, 6, and 12 months postinjury. Participants were included if they were working-age, had a GCS score of 13-15, were working pre-injury, and had a complete work history, ie, if they reported their employment status 2 weeks, 3 months, 6 months, and 12 months post-injury.

© 2022 Gaudette É et al. *JAMA Network Open*.

eTable 2. Participants' Characteristics

	Employment status known for all assessments N (%)		Significance		Weighted N (%)
	Yes	No	Unwt	Wt	Yes
Participants	435	314			435
Age					
Mean (SD)	37.3 (12.9)	36.7 (13.0)	0.227	0.25	37.5 (13.0)
Sex					
A) Male	288 (66%)	223 (71%)	0.177	0.233	291 (67%)
B) Female	147 (34%)	91 (29%)			144 (33%)
Race					
A – White	320 (74%)	258 (82%)	0.022	0.17	326 (75%)
B – Black	83 (19%)	44 (14%)			80 (19%)
C – Other	30 (7%)	12 (4%)			27 (6%)
Unknown	2	0			2
Ethnicity					
Non-Hispanic	363 (84%)	224 (71%)	<.001	0.124	350 (81%)
Hispanic	70 (16%)	90 (29%)			83 (19%)
Unknown	2	0			2
SES Employment					
1 - Working full time (35 hrs or more/week, at least minimum wage)	351 (81%)	258 (82%)	0.812	0.478	350 (80%)
2 - Working 20-34 hrs/week (at least minimum wage)	60 (14%)	36 (12%)			62 (14%)
3 - Working <20 hrs/week (at least minimum wage)	18 (4%)	15 (5%)			18 (4%)
4 - Temporary/odd jobs/less than minimum wage jobs	4 (1%)	4 (1%)			5 (1%)
5 - Special employment (sheltered workshop, supp. empl., job coach)	1 (0%)	0 (0%)			1 (0%)
Unknown	0	1			0
Income prior to injury					
1 – None	2 (1%)	3 (1%)	0.063	0.198	2 (1%)
2 - Less than $10,000	36 (10%)	37 (16%)			40 (11%)

© 2022 Gaudette É et al. *JAMA Network Open*.

3 - $10,000 to $14,999	38 (10%)	21 (9%)			38 (10%)
4 - $15,000 to $24,999	47 (13%)	36 (15%)			50 (14%)
5 - $25,000 to $34,999	58 (16%)	29 (12%)			57 (16%)
6 - $35,000 to $49,999	49 (13%)	39 (16%)			46 (13%)
7 - $50,000 to $74,999	73 (20%)	29 (12%)			69 (19%)
8 - $75,000 to $99,999	24 (6%)	17 (7%)			22 (6%)
9 - $100,000 to $149,999	27 (7%)	13 (5%)			25 (7%)
10 - $150,000 to $199,999	7 (2%)	5 (2%)			6 (2%)
11 - $200,000 or more	11 (3%)	8 (3%)			10 (3%)
Unknown	63	77			70
Education Years					
Mean (SD)	14.0 (2.8)	13.0 (2.9)	<.001	<.001	13.8 (2.8)
A - Less than high school	48 (11%)	61 (20%)			54 (12%)
C - High school only	135 (31%)	113 (36%)			146 (34%)
D - Some college	82 (19%)	63 (20%)			83 (19%)
E - 4yr degree	108 (25%)	49 (16%)			97 (22%)
F - Post-graduate	61 (14%)	26 (8%)			54 (12%)
Unknown	1	2			1
Insurance					
A) Insured	297 (69%)	188 (62%)	0.05	0.218	286 (66%)
B) Medicare/Other	44 (10%)	32 (11%)			43 (10%)
C) Uninsured	92 (21%)	83 (27%)			104 (24%)
Unknown	2	11			3
Mechanism of Injury					
A - MVC Occupant	150 (34%)	136 (43%)	0.005	0.504	160 (37%)
B – MCC	40 (9%)	32 (10%)			41 (9%)
C - MVC (cyclist or ped.)	92 (21%)	35 (11%)			80 (18%)
D – Fall	82 (19%)	61 (19%)			86 (20%)
E – Assault	26 (6%)	23 (7%)			27 (6%)
F - Other/Unknown	45 (10%)	27 (9%)			41 (9%)
ED GCS					
Mean (SD)	14.7 (0.5)	14.8 (0.5)	0.697	0.683	14.7 (0.5)
13	17 (4%)	9 (3%)			19 (4%)
14	76 (17%)	55 (18%)			78 (18%)
15	342 (79%)	250 (80%)			338 (78%)
Initial CT					
Negative	312 (72%)	220 (71%)	0.743	0.652	311 (72%)
Positive	122 (28%)	91 (29%)			123 (28%)

© 2022 Gaudette É et al. *JAMA Network Open.*

Unknown	1	3			1
Highest level of care					
1 - ED Discharge	166 (38%)	96 (31%)	0.005	0.022	159 (37%)
2 - Hospital admit no ICU	186 (43%)	133 (42%)			185 (43%)
3 - Hospital admit with ICU	83 (19%)	85 (27%)			90 (21%)

Abbreviations: SES, socio-economic status; ED, emergency department; GCS, Glasgow Coma Scale; CT, computed tomography scan; ICU, intensive care unit.
Statistical significance by Mann-Whitney and Fisher's exact test.

© 2022 Gaudette É et al. *JAMA Network Open*.

eTable 3. Symptoms at 3 Months by Work Status at 12 Months

		N	Working at 12 Months N (%) No	Yes	p	p^{BH}
Participants		435	74 (17%)	361 (83%)		
3mo Rivermead Symptom (Moderate/severe)						
Fatigue	None/Mild	312	41 (13%)	271 (87%)	0.001	0.002
	Mod/Severe	123	33 (27%)	90 (73%)		
Sleep Disturbance	None/Mild	321	41 (13%)	280 (87%)	<.001	0.001
	Mod/Severe	114	33 (29%)	81 (71%)		
Frustrated	None/Mild	321	46 (14%)	275 (86%)	0.02	0.022
	Mod/Severe	114	28 (25%)	86 (75%)		
Forgetful	None/Mild	321	42 (13%)	280 (87%)	<.001	0.001
	Mod/Severe	114	32 (28%)	81 (72%)		
Irritable	None/Mild	333	44 (13%)	289 (87%)	<.001	0.001
	Mod/Severe	102	30 (30%)	72 (70%)		
Longer to Think	None/Mild	339	44 (13%)	295 (87%)	<.001	<.001
	Mod/Severe	96	30 (32%)	66 (68%)		
Headache	None/Mild	342	46 (14%)	295 (86%)	0.001	0.001
	Mod/Severe	93	28 (30%)	66 (70%)		
Poor Concentration	None/Mild	343	44 (13%)	299 (87%)	<.001	<.001
	Mod/Severe	92	30 (33%)	62 (67%)		
Depressed	None/Mild	352	50 (14%)	302 (86%)	0.003	0.004
	Mod/Severe	83	24 (29%)	59 (71%)		
Noise Sensitivity	None/Mild	371	53 (14%)	318 (86%)	0.001	0.001
	Mod/Severe	64	21 (33%)	43 (67%)		
Restless	None/Mild	371	48 (13%)	322 (87%)	<.001	<.001
	Mod/Severe	64	26 (40%)	39 (60%)		
Dizziness	None/Mild	373	51 (14%)	322 (86%)	<.001	<.001
	Mod/Severe	62	23 (38%)	39 (62%)		
Light Sensitivity	None/Mild	386	57 (15%)	329 (85%)	0.002	0.003
	Mod/Severe	49	17 (35%)	32 (65%)		
Blurred Vision	None/Mild	393	58 (15%)	335 (85%)	0.001	0.001
	Mod/Severe	42	16 (38%)	26 (62%)		
Nausea	None/Mild	416	67 (16%)	349 (84%)	0.028	0.03
	Mod/Severe	19	7 (39%)	12 (61%)		
Double Vision	None/Mild	426	70 (16%)	356 (84%)	0.049	0.049
	Mod/Severe	9	4 (47%)	5 (53%)		
At least 1 symptom	None/Mild	209	24 (12%)	185 (88%)	0.003	0.004
	Mod/Severe	226	50 (22%)	176 (78%)		
At least 3 symptoms	None/Mild	276	31 (11%)	245 (89%)	<.001	<.001
	Mod/Severe	159	43 (27%)	116 (73%)		

Weighted analysis. Statistical significance by Fisher's exact test; BH = p-value after controlling for 5% false-discovery rate per Benjamini-Hochberg.

© 2022 Gaudette É et al. *JAMA Network Open.*

eFigure 2. Employment Status of Participants Pre-Injury and in the 12 Months After Injury (Unweighted)

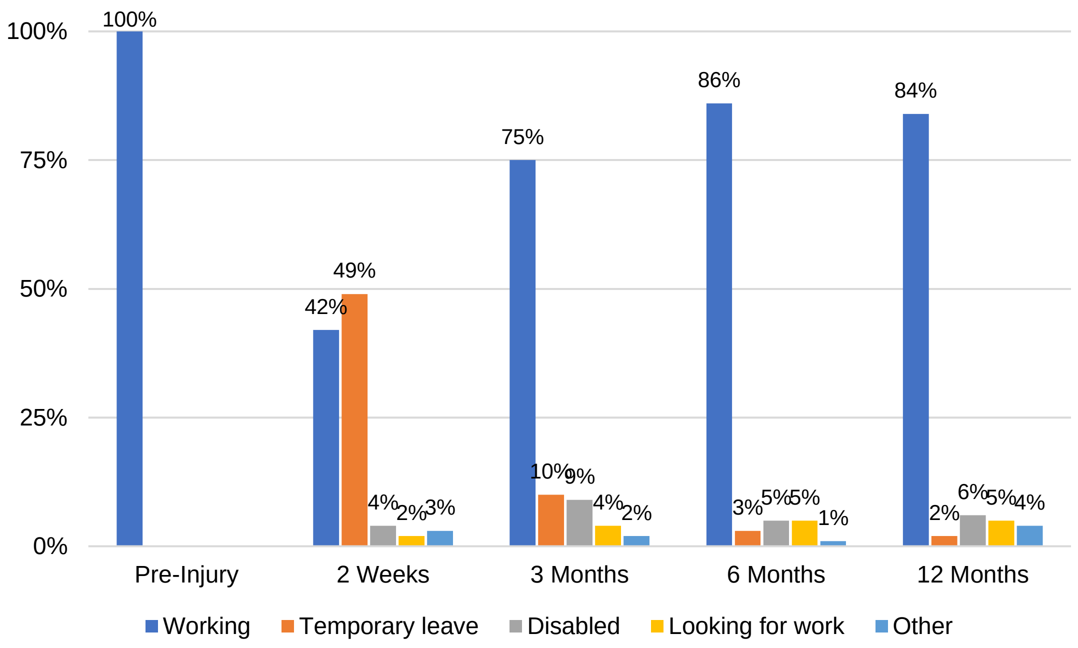

Unweighted analysis. Temporary leave status is defined as not working due to health (including maternity leave) but having a job to return to; Disabled status is defined as not working due to health and not having a job to return to; Other status includes keeping house, being a student, being retired, having an unknown status, and mentioning an unlisted status.

© 2022 Gaudette É et al. *JAMA Network Open*.

eFigure 3. Proportion of Participants Reporting Annual Income Decline by Work Status 12 Months After Injury (Unweighted)

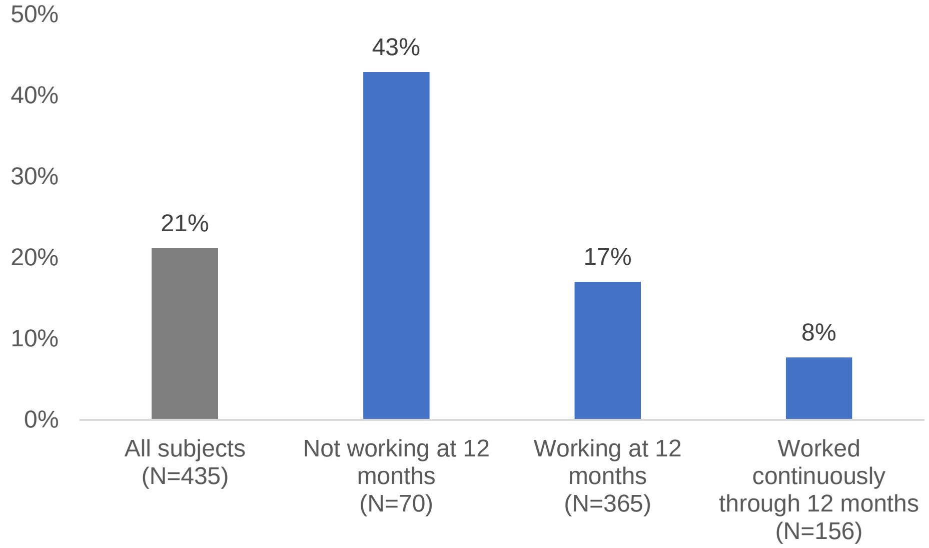

Unweighted analysis. Participants who "Worked continuously through 12 months" are the subset of participants reporting working at 12 months who also reported working 2 weeks, 3 months, and 6 months postinjury.

© 2022 Gaudette É et al. *JAMA Network Open*.

eTable 4. Work Status 12 Months After PostInjury by Baseline Injury Characteristics and Postconcussion Symptoms (Unweighted)

	N (%)	Working at 12 Months N (%)			
		No	Yes	p	P_{BH}
**Participants**	**435**	**70 (16%)**	**365 (84%)**		
**GCS**					
13-14	93 (21%)	18 (19%)	75 (81%)	.342	.436
15	342 (88%)	52 (15%)	290 (85%)		
**Admitted**					
No	166 (38%)	23 (14%)	143 (86%)	.349	.436
Yes	269 (62%)	47 (17%)	222 (83%)		
**2-Week Symptoms**					
0-2 Mod/Sev	236 (55%)	30 (13%)	206 (87%)	.035	.088
3+ Mod/Sev	194 (45%)	40 (21%)	154 (79%)		
Unknown	5	0 (0%)	5 (100%)		
**3-Month Symptoms**					
0-2 Mod/Sev	281 (65%)	30 (11%)	251 (89%)	$^{<}$.001	$^{<}$.001
3+ Mod/Sev	154 (35%)	40 (26%)	114 (74%)		

Unweighted analysis. Statistical significance by Fisher's exact test; MC = p-value after controlling for 5% false-discovery rate per Benjamini-Hochberg.

© 2022 Gaudette É et al. *JAMA Network Open*.

eTable 5. Work Status 6 and 12 Months After Injury by Employer Assistance Offered (Unweighted)

	Assistance offered by 3 months	N	Working at 6 Months					Working at 12 Months				
			N	%	Diff (%)	p	P^{MC}	N	%	Diff (%)	p	P^{MC}
All Participants		392	337	86%				328	84%			
Any assistance	No	99	76	77%				71	72%			
	Yes	293	261	89%	12%	0.004	0.007	257	88%	16%	<.001	0.001
Sick Leave	No	163	137	84%				127	78%			
	Yes	227	198	87%	3%	0.38	0.489	199	88%	10%	0.012	0.019
Modified schedule	No	228	182	80%				178	78%			
	Yes	163	155	95%	15%	<.001	<.001	150	92%	14%	<.001	0.001
Part-time / reduced hours	No	242	196	81%				189	78%			
	Yes	149	141	95%	14%	<.001	<.001	139	93%	15%	<.001	<.001
Transfer	No	355	302	85%				292	82%			
	Yes	26	25	96%	11%	0.15	0.207	26	100%	18%	0.012	0.019
Equipment/assistive technology	No	263	226	86%				219	83%			
	Yes	13	11	85%	-1%	1	1	11	85%	1%	1	1
Coaching/mentoring	No	267	229	86%				223	84%			
	Yes	9	8	89%	3%	1	1	8	89%	5%	1	1
More than one type	No	235	190	81%				182	77%			
	Yes	157	147	94%	13%	<.001	0.001	146	93%	16%	<.001	<.001

Unweighted analysis. The table is restricted to the N=392 participants who reported working at 3 months and were asked the employer assistance question. Statistical significance by Fisher's exact test; MC = p-value after controlling for 5% false-discovery rate per Benjamini-Hochberg.

© 2022 Gaudette É et al. *JAMA Network Open*.

eFigure 4. Employer Assistance Offered to Participants by 3 Months After Injury by Having Seen a Healthcare Provider (Unweighted)

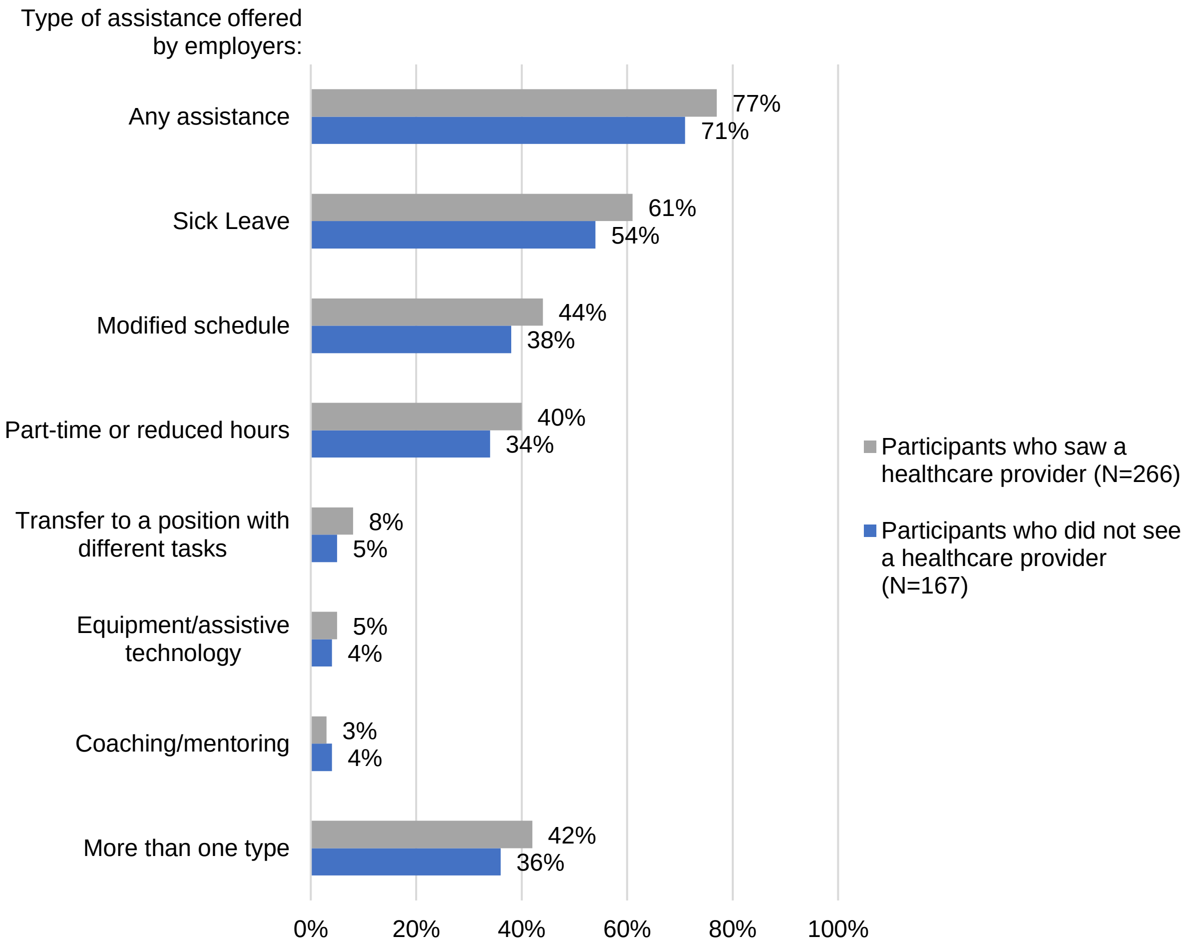

Unweighted analysis. Healthcare providers included general practitioner, brain injury/concussion clinic, neurologist, physiatrist, chiropractor, psychiatrist, psychologist, alternative medicine, or any other provider specified by the subject.

© 2022 Gaudette É et al. *JAMA Network Open*.

eTable 6. Symptoms at 3 Months by Work Status at 12 Months (Unweighted)

		N	Working at 12 Months N (%)			
			No	Yes	p	p^{MC}
**Participants**		**435**	**70 (16%)**	**365 (84%)**		
**3mo Symptom**						
Headache	None/Mild	345	44 (13%)	301 (87%)	0.001	0.001
	Mod/Severe	90	26 (29%)	64 (71%)		
Dizziness	None/Mild	378	48 (13%)	330 (87%)	<.001	<.001
	Mod/Severe	57	22 (39%)	35 (61%)		
Nausea	None/Mild	417	64 (15%)	353 (85%)	0.053	0.053
	Mod/Severe	18	6 (33%)	12 (67%)		
Noise Sensitivity	None/Mild	372	50 (13%)	322 (87%)	0.001	0.001
	Mod/Severe	63	20 (32%)	43 (68%)		
Sleep Disturbance	None/Mild	323	39 (12%)	284 (88%)	<.001	0.001
	Mod/Severe	112	31 (28%)	81 (72%)		
Fatigue	None/Mild	314	39 (12%)	275 (88%)	0.001	0.002
	Mod/Severe	121	31 (26%)	90 (74%)		
Irritable	None/Mild	335	41 (12%)	294 (88%)	<.001	<.001
	Mod/Severe	100	29 (29%)	71 (71%)		
Depressed	None/Mild	353	47 (13%)	306 (87%)	0.002	0.003
	Mod/Severe	82	23 (28%)	59 (72%)		
Frustrated	None/Mild	323	43 (13%)	280 (87%)	0.011	0.012
	Mod/Severe	112	27 (24%)	85 (76%)		
Forgetful	None/Mild	326	40 (12%)	286 (88%)	<.001	0.001
	Mod/Severe	109	30 (28%)	79 (72%)		
Poor Concentration	None/Mild	345	41 (12%)	304 (88%)	<.001	<.001
	Mod/Severe	90	29 (32%)	61 (68%)		
Longer to Think	None/Mild	342	41 (12%)	301 (88%)	<.001	<.001
	Mod/Severe	93	29 (31%)	64 (69%)		
Blurred Vision	None/Mild	396	55 (14%)	341 (86%)	<.001	0.001
	Mod/Severe	39	15 (38%)	24 (62%)		
Light Sensitivity	None/Mild	388	55 (14%)	333 (86%)	0.005	0.006
	Mod/Severe	47	15 (32%)	32 (68%)		
Double Vision	None/Mild	427	66 (15%)	361 (85%)	0.026	0.027
	Mod/Severe	8	4 (50%)	4 (50%)		
Restless	None/Mild	372	45 (12%)	327 (88%)	<.001	<.001
	Mod/Severe	63	25 (40%)	38 (60%)		
At least 1 symptom	None/Mild	213	23 (11%)	190 (89%)	0.004	0.005
	Mod/Severe	222	47 (21%)	175 (79%)		
At least 3 symptoms	None/Mild	281	30 (11%)	251 (89%)	<.001	<.001
	Mod/Severe	154	40 (26%)	114 (74%)		

Unweighted analysis. Statistical significance by Fisher's exact test; MC = p-value after controlling for 5% false-discovery rate per Benjamini-Hochberg.

© 2022 Gaudette É et al. *JAMA Network Open.*
