# Supplementary material for: Employment and Economic Outcomes of Participants With Mild Traumatic Brain Injury in the TRACK-TBI Study
Source: JAMA Netw Open. 2022 Jun 29;5(6):e2219444. doi: 10.1001/jamanetworkopen.2022.19444 (PMC9244609; doi:10.1001/jamanetworkopen.2022.19444)
Supplement: Supplement 2. — Nonauthor Collaborators [file jamanetwopen-e2219444-s002.pdf]

Supplemental Online Content: Nonauthor Collaborators

*First name, last name, and suffix (if applicable) are required and will appear in PubMed.

| *Group Name(s): The TRACK-TBI Investigators | | | | | | | |
|---|---|---|---|---|---|---|---|
| ***First Name and Middle Initial(s)** | ***Last Name** | ***Suffix (eg, Jr, III)** | Academic Degrees | Institution | Location (city, state/province, country) | Role or Contribution, eg, chair, principal investigator | Group (if more than 1 Group listed in the byline) and/or Subgroup (eg, Steering Committee) |
| Neeraj | Badjatia | | MD | University of Maryland | Baltimore, MD, USA | Principal Investigator | |
| Ann-Christine | Duhaime | | MD | MassGeneral Hospital for Children | Boston, MA, USA | Principal Investigator | |
| Adam | Ferguson | | PhD | University of California, San Francisco | San Francisco, CA, USA | Principal Investigator | |
| Shankar | Gopinath | | MD | Baylor College of Medicine | Houston, TX, USA | Principal Investigator | |
| Ramesh | Grandhi | | MD | University of Utah | Salt Lake City, UT, USA | Principal Investigator | |
| Ruchira | Jha | | MD MSc | Barrow Neurological Institute | Phoenix, AZ, USA | Principal Investigator | |
| C Dirk | Keene | | MD PhD | University of Washington | Seattle, WA, USA | Principal Investigator | |
| Ryan | Kitagawa | | MD | UT Houston | Houston, TX, USA | Principal Investigator | |
| Christine | Mac Donald | | PhD | University of Washington | Seattle, WA, USA | Principal Investigator | |
| Christopher | Maddedn | | MD | UT Southwestern | Dallas, TX, USA | Principal Investigator | |
| Mike | McCrea | | PhD | Medical College of Wisconsin | Milwaukee, WI, USA | Principal Investigator | |
| Pratik | Mukherjee | | PhD | University of California, San Francisco | San Francisco, CA, USA | Principal Investigator | |
| Laura | Ngwenya | | MD PhD | University of Cincinnati | Cincinnati, OH, USA | Principal Investigator | |
| David | Okonkwo | | MD PhD | University of Pittsburgh | Pittsburgh, PA, USA | Principal Investigator | |
| Claudia | Robertson | | MD | Baylor College of Medicine | Houston, TX, USA | Principal Investigator | |
| Eric | Rosenthal | | MD | Massachusetts General Hospital | Boston, MA, USA | Principal Investigator | |
| Andrea | Schneider | | MD PhD | University of Pennsylvania | Philadelphia, PA | Principal Investigator | |
| David | Schnyer | | PhD | UT Austin | Austin, TX, USA | Principal Investigator | |
| Murray | Stein | | MD MPH | University of California, San Diego | San Diego, CA, USA | Principal Investigator | |
| Sabrina | Taylor | | PhD | University of California, San Francisco | San Francisco, CA, USA | Clinical Trial Manager | |
| Mary | Vassar | | RN MS | University of California, San Francisco | San Francisco, CA, USA | Data Manager | |
| John | Yue | | MD | University of California, San Francisco | San Francisco, CA, USA | Resident Physician | |
